# Case Report of Urethritis in a Male Patient Infected with Two Different Isolates of Multiple Drug-Resistant *Neisseria gonorrhoeae*

**DOI:** 10.3389/fmed.2017.00194

**Published:** 2017-11-08

**Authors:** Lamiaa Al-Madboly, Shereen Gheida

**Affiliations:** ^1^Faculty of Pharmacy, Department of Pharmaceutical Microbiology, Tanta University, Tanta, Egypt; ^2^Faculty of Medicine, Department of Dermatology and Venereology, Tanta University, Tanta, Egypt

**Keywords:** urethritis, conjunctivitis, arthritis, *Neisseria gonorrhoeae*, resistance

## Abstract

We report a brief description of a case suffering from bacterial urethritis, conjunctivitis, and arthritis, caused by two different isolates of multiple drug-resistant *Neisseria gonorrhoeae*. Initial diagnosis was dependent on the patient history, clinical findings, symptoms, and the bacteriological data. Polymerase chain reaction confirmed the identification of the pathogens. Random amplified polymorphic DNA revealed two different patterns. Susceptibility testing was performed using Kirby–Bauer disk diffusion method and the minimum inhibitory concentration was also determined. It revealed multiple drug resistance associated with β-lactamase production. Only gentamicin, rifampicin, and azithromycin were active against the test pathogens. A dual therapy was initiated using gentamicin as well as azithromycin to treat the possible co-infection with *Chlamydia trachomatis*. Complete recovery of the patient achieved with resolved symptoms a week later.

## Background

Sexually transmitted diseases cause high rates of morbidity and even mortality in adults and newborns. Many of them amplify the risk of HIV acquisition. In the developing countries, incidence and prevalence of sexually transmitted diseases are high, constituting a substantial health and economic burden, particularly for countries strained with other emerging health problem (1).

Gonorrhea is one of the most common sexually transmitted diseases. It is recognized as acute bacterial infection, which commonly transmitted through sexual contact or perinatal. The patient may be asymptomatic, or suffering from urethritis, arthritis, conjunctivitis, cervical, pelvic inflammatory disease, and bacteremia (2).

The majority of infectious urethritis cases in men is caused by sexually transmitted diseases, which may be gonococcal (gonorrhea) or non-gonococcal (chlamydia). These diseases are characterized by urethral inflammation in the form of discharge, dysuria, and burning upon urination. Few days later, purulent and bloody discharge may be present. Many complications including prostatitis, epididymitis, proctitis, and infertility have been previously reported. However, the patient may be asymptomatic in some cases leading to a carrier state (3). Bacteremia usually causes disseminated gonococcal infection that appears in the form of fever, oligoarthritis, and polyarthralgia. Neonates may be infected by gonococci during their passage through infected birth canal resulting in bilateral conjunctival inflammation known as “ophthalmia neonatorum.”

The causative agent of gonnorrhoea is a bacterium termed *Neisseria gonorrhoeae*. This pathogen is Gram-negative non-motile aerobic diplococcus. It is fastidious microorganism, which requires incubation in the presence of CO_2_. Multiple virulence factors, which strongly participate in the pathogenesis of gonococcal infections, have been documented. Gonococci initially adhere to the epithelial cells followed by penetration until reach the subepithelial space where infection is established. Gonococcal pili, porin, and Opa proteins mediate adhesion and invasion. In addition, the presence of lipopolysaccharides provoke an inflammatory response and result in TNF-α release which is responsible for the majority of the symptoms accompanying the disease. Furthermore, stimulation of huge number of neutrophil may lead to microabscesses (4).

For rapid diagnosis of *N. gonorrhoeae*, microscopic examination of Gram- or methylene blue-stained urethral smear is carried out but culture is still of great importance to perform a susceptibility test. In this work, we report a case of urethritis caused by two different isolates of multiple drug-resistant *N. gonorrhoeae* in a male patient.

## Case Report

A 26-year-old male patient came to the dermatology clinic of Tanta University hospital complaining from severe burning sensation during urination and dysuria for 4 days. Additionally, he was suffering from penile discharge and testicular tenderness. He had a history of multiple heterosexual relationships with a last contact 8 days ago. On physical examination, vital signs showed: blood pressure 110/79, pulse 75, and temperature 37.6°C. There was mucopurulent cloudy discharge from urethra. Swollen testicles were also observed. When the patient asked about any other symptoms, he mentioned feeling fatigue with pain in the knee joints and ankles 2 weeks ago but he did not receive any medical remedy until the appearance of severe irritation, redness in the eye, as well as edema in the eyelid with the presence of copious discharge (conjunctivitis). These symptoms seem to be unrelated to a degree that may obscure the diagnosis.

**Figure 1:**
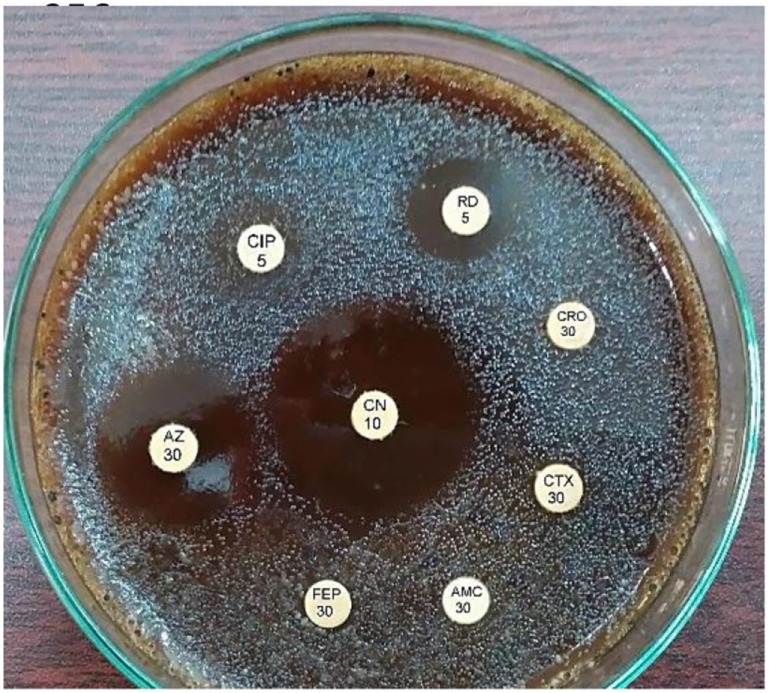
Susceptibility test using Kirby–Bauer disk diffusion method.

**Figure 2:**
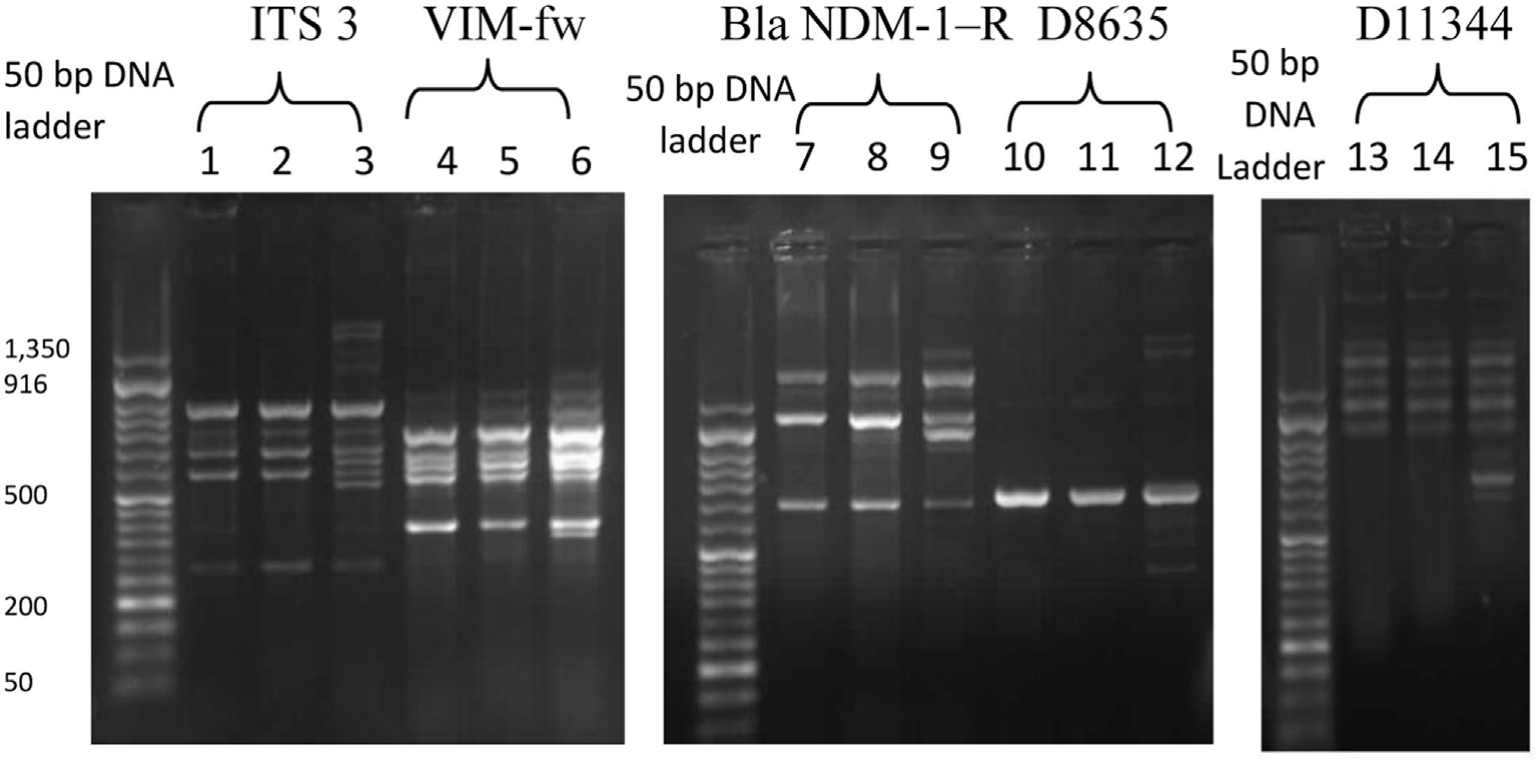
The random amplified polymorphic DNA polymerase chain reaction fingerprint pattern for three *Neisseria gonorrhoea* strains isolated from urethral discharge (lanes 1, 4, 7, 10, 13), ocular swab (lane 2, 5, 8, 11, 14), and blood (lane 3, 6, 9, 12, 15) using five sets of arbitrary primers including IT3, VIM-f, Bla NDM-1R, D8635, and D11344. DNA Ladder: 50 bp.

Following counseling, urethral and ocular swabs, and blood sample were aseptically obtained and streaked immediately on Thayer Martin and chocolate agar plates then incubated overnight at 37°C in the presence of 5% CO_2_. Following the incubation period, Grayish white, transparent to opaque, slightly raised colonies with 1–2 mm diameter were observed. After Gram-staining, pink to red diplococci with coffee bean-shaped cells opposing each other on the concave sides. This result was sufficient for the presumptive identification of *N. gonorrhoeae*. Furthermore, numerous polymorphonuclear cells with intracellular diplococci, were microscopically detected in the urethral smear. Cytochrome C oxidase was tested using the commercially available strips (Biolife, Italy) impregnated with *N,N,N′,N′* tetramethyl-p-phenylenediamine dihydrochloride and dried. A deep blue color appeared within 30 s indicating positive result. The test pathogens were also identified by the matrix assisted laser desorption-ionization time-of-flight (MALDI-TOF) in the Microbiology department, Faculty of Medicine, Alexandria University using Bruker Daltonik MALDI Biotyper, Germany. For confirmation, polymerase chain reaction (PCR) was utilized to amplify the pathogenic DNA in the presence of specific primers. Amplicons with 390 bp were detected in the electrophoregram. Using Kirby–Bauer disk diffusion method, susceptibility testing of the recovered isolates was performed against different antimicrobials. *N. gonorrhoea* ATCC 49226 was used as a quality control strain. The results of susceptibility testing were interpreted according to CLSI (5). It revealed multiple drug resistance to ampicillin, ampicillin/clavulanic acid, cephradine, cefotaxime, cefepime, cefuroxime, ceftriaxone, ciprofloxacin, chloramphenicol, sulfamethoxazole, trimethoprim, tetracycline, doxycycline, and spectinomycin. Only gentamicin, rifampicin, and azithromycin were active against the test pathogen (Figure 1). The minimum inhibitory concentration (MIC) was determined in order to calculate the fold increase in resistance (Table 1). It was noted that the resistance patterns of the three strains isolated from the three body sited were the same. β-lactamase production was detected using nitrocefin test. The yellow color of nitrocefin solution (Oxoid, England) was quickly turned into red indicating positive result. Random Amplified Polymorphic DNA (RAPD) was performed using different arbitrary primers including; ITS3 (5′-GCATCGATGAAGAACGCAGC), VIM-fw (5′-GTACGCATCACCGTCGACAC), Bla _NDM_-1 -R GTAGTGCTCAGTGTCGGCAT D8635 (5′-GAGCGGCCAAAGGGAGCAGAC), D11344 (5′-AGTGAATTCGCGGTGAGATGCCA) (6, 7) in order to detect if the three strains isolated from different body sites were the same or different. It revealed identical patterns for both strains isolated from urethral discharge and ocular swab suggesting autoinfection through the accidental contamination of the eyes by urethral discharge. However, the strain isolated from blood showed different pattern confirming that pathogenesis was due to two different strains of *N. gonorrhoeae* (Figure 2). This might explain the early appearance of arthritis symptoms. A dual therapy was initiated with 240 mg of gentamicin administered as intravenous infusion over 30 min as well as azithromycin (2 g as a single oral dose) to treat the possible co-infection with *Chlamydia trachomatis*. Normal saline solution was used to irrigate the patients’ eyes four times per day. Complete recovery of the patient achieved with resolved symptoms a week later.

**Table 1:** The minimum inhibitory concentrations (MICs) of different antimicrobials tested against *N. gonorrhoeae* strains.

Antimicrobial agents	MICs (μg/mL) against *N. gonorrhea* strains			Fold increase in MIC of the test relative to the QC strain
	ATCC 49226	Isolate from urethral discharge/conjunctiva	Isolate from blood	
Ampicillin	0.03	64	64	2,133.3
Ampicillin/clavulanic acid	0.03	64	16	533–2,133
Cephradine	0.25	64	16	128–256
Cefotaxime	0.25	16	16	64
Cefipime	0.25	16	16	64
Cefuroxime	0.25	16	16	64
Ceftriaxone	0.008	32	16	2,000–4,000
Ciprofloxacin	0.008	32	16	4,000–2,000
Tetracycline	0.25	32	32	128
Doxycycline	0.25	16	16	64
Chloramphenicol	0.03	128	128	4,267
Sulfamethoxazole	0.25	128	128	512
Trimethoprim	0.25	128	128	512
Spectinomycin	4	256	64	16–64
Gentamicin	0.001	0.06	0.06	–
Azithromycin	0.125	0.25	0.25	–
Rifampicin	0.001	0.06	0.06	–

## Discussion

Despite the significant progress in the diagnosis, surveillance, and treatment protocols, gonorrhea is still one of the most important sexually transmitted diseases that can lead to urethritis in men (8). According to World Health Organization, *N. gonorrhoeae* infect sexually active individuals of age range 15–50 years and thus creating a serious public health problem (9, 10).

In Egypt, there is lack of the information available about sexually transmitted diseases. However, Ali et al. (11) reported that the prevalence of sexually transmitted disease was increasing day by day. Little was known about the actual prevalence and incidence of these diseases in Egypt. Out of the 95 cases studied by Ali et al. (11), there were 68 (71.6%) positive cases of sexually transmitted diseases. *N. gonorrhoeae* isolates were recovered from approximately 37% of the positive cases. Additionally, the study of Elkayal et al. (12) determined the prevalence of *N. gonorrhoeae* among infertile female patients and they reported that the positive cases were 3 out of 79 suspected cases (3.8%). Amin (1) stated that the epidemiological data of sexually transmitted diseases in Egypt are extracted from intermittent researches. The impact of these diseases on the public health is largely undefined due to poor surveillance system as well as the poor access to the reproductive health information.

*Neisseria gonorrhoeae* is recognized as strict human pathogen that commonly infects the urogenital tract including urethra of male and endocervix of reproductive female. Colonization of rectum, conjunctiva, and pharynx may be involved (13). Hematogenic spread may facilitate the gonococcal distribution to the joints and skin (14). *N. gonorrhoeae* could facilitate HIV acquisition. In response to gonococcal infection, genital epithelial cells can produce cytokines, chemokines and defensins to modulate HIV infection. The number of endocervical CD4+ T cells (HIV target) had been increased in gonococcal infected women and hence increased the risk of acquiring HIV (15, 16). Gonococcal infections usually asymptomatic in female while symptomatic in male patients. Therefore, symptoms appear on male patients infected with *N. gonorrhoeae* increase the probability of early diagnosis and hence curative treatment preventing the sequelae (17). *C. trachomatis* is an obligate intracellular human pathogen accounting for most of the cases of sexually transmitted diseases and preventable blindness in the world. Prevalence of chlamydia infection is highest in persons aged ≤24 years. Asymptomatic chlamydial infection is common among both men and women. Mucosal surfaces of urogenital system and conjunctivae are the primary target of this pathogen in the human body (17). The study of Zigangirova et al. (18) reported that 59.6% of serum specimens were tested positively in culture test suggesting that extragenital chlamydial complications may be associated with systemic spread of infection. About 10–30% of the sexually infected patients were co-infected with *C. trachomatis* and *N. gonorrhoeae* and hence should be treated for chlamydia synchronously (19).

Gram staining of the urethral smear provides sufficient presumptive diagnosis of *N. gonorrhoeae* infection. However, specific diagnosis could reduce the probable complications. Nucleic acid amplification tests (NAATs) are important to confirm the identification of *N. gonorrhoeae*. Treatment of gonnorrhoea is complicated due to the ability of the causative pathogen to develop resistance against antimicrobials resulting in treatment failure so that clinicians are advised to do a sensitivity test. Consequently, culture is necessary for doing a susceptibility test before therapy initiation (20).

While female patients suffering from gonococcal infections are mostly asymptomatic, male patients commonly present dysuria accompanied with urethral discharge as a major symptom. Inflammation of the conjunctiva due to gonococci is a rare symptom in adult patients and usually caused by autoinfection through the accidental transfer of the urethral discharge into the eyes. This kind of infection is devastating to the eye that may cause corneal perforation and loss of vision. Patients usually fail to relate the mucopurulent ocular discharge with their genito-urinary complain that may be missed out resulting in diagnosis failure. Haemophilus species, *C. trachomatis*, and viruses are other causes of mucopurulent conjunctivitis should be considered unless there is a sexual behavior history. Gonococcal arthritis involves joints at different sites in the body including; hands, fingers, and feet but spine does not affected (21–23). In the present case, the patient had a history of heterosexual contact 8 days prior to the onset of symptoms appearance. He had developed certain symptoms such as purulent urethral discharge with dysuria, burning sensation during micturition, conjunctivitis, and arthritis. The initial diagnosis was dependent on the patient history, clinical findings, symptoms, and the bacteriological data (colony morphology, Gram stain result, and the biochemical tests). PCR confirmed the identification of the pathogen. RAPD PCR technique is considered as a highly discriminating typing method for *N. gonorrhoeae*. It revealed two different patterns reflecting two different strains participated in the pathogenesis. Mbuagbaw et al. (24) reported similar cases.

Recently, penicillins, sulfonamides, tetracyclines, spectinomycin, fluoroquinolones, macrolides, and cephalosporin-resistance have been reported in several countries (25). The emergence of multidrug (MDR) or extensively drug-resistant (XDR) gonococcal strains is attributed to the acquisition of multiple antimicrobial resistance traits. With regard to gonorrhea, MDR was defined as acquired non-susceptibility to at least one agent in three or more antimicrobial categories (26). Additionally, Trembizki et al. (27) reported that *N. gonorrhoeae* isolates harbored a gene encoding for penicillinase production resulting in the spread of penicillin resistance among gonococci through horizontal gene transfer. In the present case report, production of β-lactamase by the test pathogen was confirmed and this might be contributed to the development of penicillin- and cephalosporin-resistance.

Uncomplicated gonococcal urethritis or that associated with conjunctivitis treated by 1 g of ceftriaxone given intramuscularly as a single dose (24, 28). The eyes usually irrigated by normal saline four times daily. Topical antibiotics sometimes involved in the treatment (29). For complicated gonococcal infection, the patient received 1 g of ceftriaxone either intravenously or intramuscularly twice a day for 48 h then the dose reduced to 1 g per day for 5 days. One gram of azithromycin should be prescribed due to the possible co-infection with *C. trachomatis* (24). Cefotaxime, ciprofloxacin, spectinomycin, oflxacin, and cefixime were also recommended unless there was emerging drug resistance such as the present case. For patients infected with cephalosporin-resistant *N. gonorrhoeae*, 240 mg of gentamicin was IV infused over 30 min. This alternative antibiotic usually combined with 2 g of azithromycin in a single oral dose. The later antimicrobial agent should not be prescribed as a monotherapy due to reported resistance (30). Based on the culture susceptibility test as well as the treatment guidelines of gonorrhea (30), we followed the later regimen with good outcome. It is recommended that all patients who have been treated with the alternative treatment regimen should undergo a cure test using culture after 3–7 days of treatment completion. It is preferable to repeat the examination of the patient after 6 months of treatment (30). In the present case, bacterial culture was repeated 1 week after recovery and showed negative result. Six months later, another culture was tested with no bacterial growth.

## Conclusion

Patients with risky sexual behavioral history presented with copious mucopurulent urethritis associated with conjunctivitis and painful joints suspected in a high index to be infected with *N. gonorrhoeae*. Co-infection with *C. trachomatis* could not be excluded and hence should be covered in the treatment regimen. Bacteriology and NAAT had great roles in the definitive diagnosis as well as the follow-up. RAPD PCR was able to discriminate between different strains isolated from different body site. Susceptibility tests were far important that helped in the success of the therapy.

## Ethics Statement

Written informed consent was obtained from the patient to participate in the present study as well as for the publication of this case report. The Ethics Committee of Tanta University Hospital already approved this work.

## Author Contributions

LA-M and SG contributed to this work; revised and approved the paper. SG examined the patient and recorded the signs and symptoms. LA-M conducted the microbiological and molecular tests; wrote the paper.

## Conflict of Interest Statement

The authors declare that the research was conducted in the absence of any commercial or financial relationships that could be construed as a potential conflict of interest.
